# 1035. The Microbiological Pattern and Outcome of Community Acquired Brain Abscess: A Single Centre Study from Pakistan

**DOI:** 10.1093/ofid/ofac492.876

**Published:** 2022-12-15

**Authors:** Muneeba A Sayeed

**Affiliations:** Sindh Infectious Diseases Hospital & Research Center/Dow University of Health Sciences, Karachi, Sindh, Pakistan

## Abstract

**Background:**

Brain abscess is a potentially life-threatening infection. Streptococci and bacteroides are considered the predominant causative organisms in community acquired brain abscesses. Recommended empirical therapy is therefore ceftriaxone and metronidazole. Our objective was to assess the microbiological pattern and outcome in patients admitted with brain abscess in the neurosurgery unit of a tertiary care hospital in Karachi, Pakistan.

**Methods:**

A retrospective study was conducted on all patients admitted with community acquired brain abscess at the Neurosurgery Department of Shaheed Mohtarma Benazir Bhutto Institute of Trauma from Jun 2018- Nov 2019. Demographic, microbiological, management and outcome data was gathered from medical records.

**Results:**

Twenty-three patients were identified of which 13 were males (56.5%). Mean age was 18 (2-65) years. Predisposing factor was an otogenic focus in 17 (73.9%), and head trauma in 3 (13%). Of 23 patients, site of abscess was frontal in 7 (30.4%), occipital in 8 (34.7%), parietal in 4 (17.3%), and temporal in 3 (13%). Single brain abscess was seen in 18 (78.2%). Abscess was drained in all 23 patients (100%) patients and pus culture positive in 16, of which 8 (50%) were poly-microbial. Isolates were 6 (%) Bacteroides, 6 Streptococci, 5 Enterobacteriace, 3 Staphylococcus *aureus*, 3 Enterococci, 1 Hemophilus, 1 Acinetobacter and 1 Brevibacterium. Of 6 bacteroides isolates, 2 were resistant to metronidazole while only 3 of 10 aerobes isolated were susceptible to ceftriaxone. All patients did well with resolution of abscess/es after an average of 41.2 days of therapy.

**Figure:**
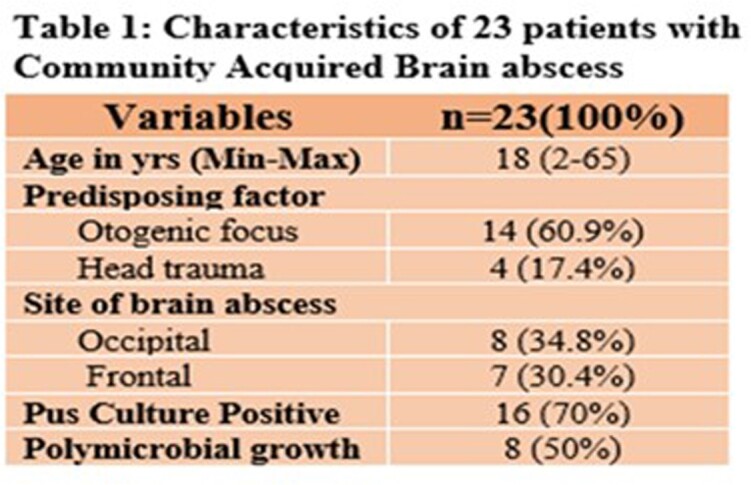

**Figure:**
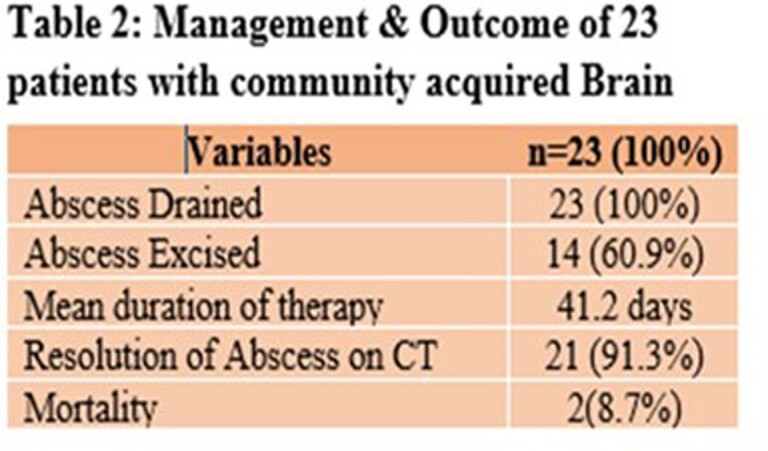

**Figure:**
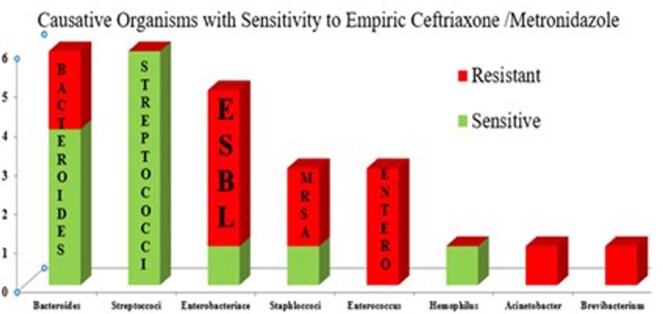

**Conclusion:**

The microbiological spectrum and antimicrobial susceptibility pattern of community acquired brain abscess is changing. Ceftriaxone and metronidazole may no longer be appropriate empirical therapy in these patients.

**Disclosures:**

**All Authors**: No reported disclosures.

